# Low-dose cone-beam CT for gastric volumetry in endoscopic sleeve gastroplasty

**DOI:** 10.1186/s41747-025-00637-3

**Published:** 2025-10-07

**Authors:** Ilaria Nacci, Roberto Moretti, Andrea Morasca, Valerio Pontecorvi, Cristiano Spada, Evis Sala, Ivo Boškoski

**Affiliations:** 1 Dipartimento di Diagnostica per Immagini e Radioterapia Oncologica, Fondazione Policlinico Universitario Agostino Gemelli IRCCS, Rome, Italy; 2 Advanced Radiology Center, Fondazione Policlinico Universitario Agostino Gemelli IRCCS, Rome, Italy; 3 Dipartimento di Diagnostica per Immagini e Radioterapia Oncologica, UOC Fisica per le Scienze della Vita, Fondazione Policlinico Universitario Agostino Gemelli IRCCS, Rome, Italy; 4 Digestive Endoscopy Unit, Fondazione Policlinico Universitario Agostino Gemelli IRCCS, Rome, Italy

**Keywords:** Bariatric medicine, Cone-beam computed tomography, Gastroplasty, Radiation dosage, Stomach

## Abstract

**Abstract:**

The study aimed to evaluate the feasibility of using cone-beam computed tomography (CBCT) for gastric volume assessment before and after endoscopic sleeve gastroplasty (ESG), focusing on patient radiation dose. Ten patients scheduled for ESG were prospectively enrolled. Each patient underwent three CBCT scans under anesthesia: two scans following CO_2_ insufflation of the gastric lumen—one pre-ESG and one post-ESG—and a third scan post-ESG after gastric distension with Gastrografin. Image quality was evaluated for its adequacy in generating three-dimensional reconstructions and calculating gastric volumes. Dose-area product was recorded for each scan and used to estimate the effective dose (ED) via Monte Carlo simulations using the PCXMC rotational model. Although image quality did not match conventional computed tomography (CT), it was sufficient for three-dimensional reconstruction and gastric volume measurements. The median ED was 4.2 mSv for pre-ESG scans with CO_2_ insufflation, 4.2 mSv for post-ESG scans with CO2 insufflation, and 4.8 mSv for post-ESG scans with Gastrografin. CBCT provided satisfactory image quality for gastric volumetry at relatively low radiation doses, with ED being approximately 50% of that of conventional CT. This preliminary feasibility study suggests that CBCT could be a useful tool for planning ESG and assessing post-procedural outcomes.

**Relevance statement:**

Low-dose CBCT provided sufficient image quality for gastric volumetry in a small cohort of patients undergoing ESG, reducing radiation exposure by approximately 50% compared to conventional CT. This investigational technique enables seamless intraoperative imaging that could improve planning and evaluation of endoscopic bariatric procedures.

**Key Points:**

- CBCT allowed gastric volumetric assessment at a relatively low radiation dose.
- Scans with CO_2_ insufflation delivered lower radiation doses than scans with Gastrografin.
- Scans with CO_2_ insufflation showed superior image quality compared to scans with Gastrografin.
- CBCT could be a valuable tool for planning ESG and evaluating outcomes.

**Graphical Abstract:**

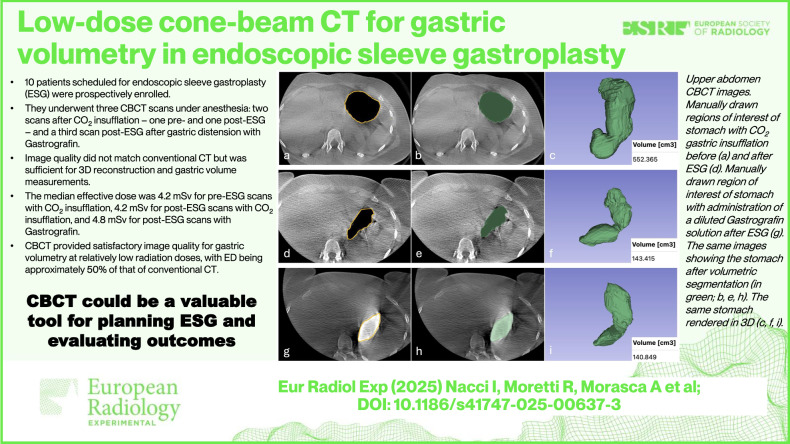

## Introduction

Obesity is associated with significant comorbidities and an increased risk of mortality compared to individuals with a normal weight. Therefore, the treatment and prevention of obesity is considered a high priority [1]. Endoscopic sleeve gastroplasty (ESG) is a minimally invasive procedure designed to induce weight loss by restricting gastric volume, providing a valid nonsurgical alternative to gastric sleeve surgery and similar bariatric surgery procedures [2–5]. However, even when technically successful, ESG may not provide lasting results as suture loss can occur, reversing the procedure and leading to weight regain [6].

Although gastric volume measurement following sleeve gastrectomy is not routinely performed, it can provide valuable insights. Gastric volume in the early postoperative period may correlate with long-term weight loss outcomes and offer surgeons feedback for optimizing the gastric sleeve creation [7–9]. In this context, imaging plays a critical role, providing precise anatomical information for procedure planning and enabling gastric volumetry to evaluate patients with unsatisfactory outcomes immediately after the procedure and during follow-up [10].

While low-cost and widely available, conventional radiographic studies with biplanar images offer limited anatomical detail and are unsuitable for accurate gastric volumetry [7]. Conventional computed tomography (CT) enables three-dimensional (3D) gastric volumetry and can be performed using low-dose protocols. However, transportation of the patient to a CT room before and after the ESG procedure is needed. Furthermore, standard CT systems often have limitations, including a typical gantry diameter of 70 cm, which can restrict their use in patients with severe obesity [11].

Cone-beam CT (CBCT), performed using angiographic systems, offers additional advantages. It allows for further reductions in radiation dose while maintaining sufficient image quality for visualizing anatomical structures. CBCT systems also provide greater flexibility in patient positioning, support table loads of up to 280 kg, and enable volumetric imaging directly within the interventional suite. This eliminates the need for patient transportation and allows both pre- and post-procedural imaging to be conducted seamlessly [12, 13].

No studies have provided objective pre- and post-procedural measurements of the gastric lumen using CBCT or examined the associated radiation exposure.

In line with the ALARA (As Low As Reasonably Achievable) principle, imaging should achieve sufficient quality to meet diagnostic needs while minimizing radiation exposure [14]. By this principle and considering the additional advantages, the primary aim of our study was to evaluate the feasibility of using CBCT for calculating gastric volumetry in patients undergoing ESG, by assessing scans acquired before and after the procedure, with both CO2 gastric insufflation and administration of a diluted Gastrografin solution. The secondary objective was to investigate the clinical radiation dose associated with CBCT scans of the upper abdomen in comparison to the dose of conventional CT.

## Materials and methods

### Participants

This single-center prospective study was conducted in accordance with the ethical principles outlined in the Declaration of Helsinki and adhered to the recommendations of Good Clinical Practice guidelines. The study received approval from the ethics committee (Prot N. 0028394/23; October 5, 2023).

Eligible participants were identified from patients referred to the Obesity Clinic at the Fondazione Policlinico Universitario Agostino Gemelli IRCCS in Rome, Italy, between February 2024 and January 2025. Only patients with a clinical indication for ESG were included, and the procedure was performed per routine clinical practice. Study participants were provided with written information and an explanation regarding the study. Patients who agreed to participate provided written informed consent.

The inclusion criteria were as follows: (a) age between 20 years and 65 years; and (b) body mass index (BMI) between 30 kg/m² and 45 kg/m². Exclusion criteria included: (a) prior surgery involving the stomach; (b) organic or motility disorders of the stomach; (c) severe systemic illnesses, including chronic kidney disease and inflammatory bowel disease; and (d) anticoagulant treatment and/or coagulopathy.

Individual body weight and height were recorded on the day of the intervention.

### ESG procedure and image acquisition

The ESG procedure was performed in a hybrid operating room equipped with an angiographic system (ARTIS Pheno, Siemens Healthcare GmbH). Patients underwent general anesthesia with single-lumen endotracheal tube intubation before the procedure, consisting of endoscopic suturing of the gastric wall extending from the antrum to the fundus [3]. The suturing technique not only reduced the diameter of the stomach, creating a tubular lumen, but also shortened its length, creating an “accordion” effect (Supplementary Fig. S1).

Each patient underwent upper abdominal CBCT acquisitions performed with the ARTIS Pheno, using a rotational protocol covering a 360° circular trajectory (“7 s Large Volume 360°” mode).

**Fig. 1:**
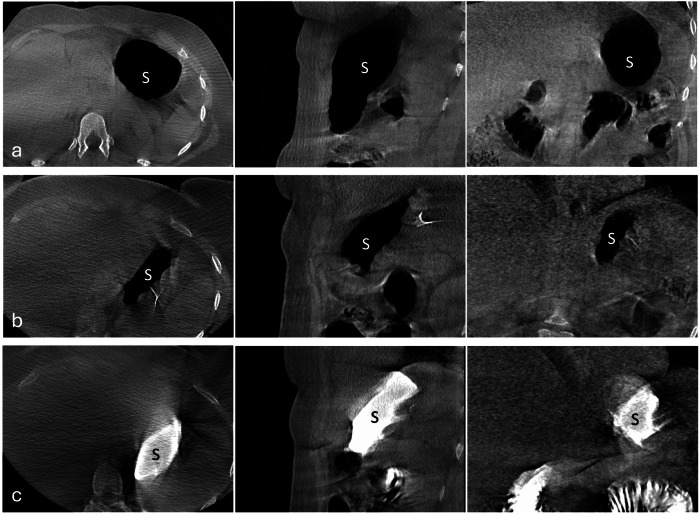
Volumetric CBCT (axial, sagittal and coronal planes, shown from left to right) acquired in the patient’s upper abdomen with CO_2_ gastric insufflation before (**a**) and after (**b**) the ESG procedure; volumetric CBCT (axial, sagittal and coronal planes, and shown from left to right) acquired with administration of a diluted Gastrografin solution after the ESG procedure (**c**). CBCT, Cone-beam computed tomography; S, Stomach

For each patient, three CBCT scans were acquired. Two scans were obtained after insufflation of CO_2_ into the gastric lumen—one before and one after the ESG procedure. A third scan, performed after ESG, was acquired following gastric distension using diluted Gastrografin to enhance visualization—prepared in a 1:3 ratio of Gastrografin to physiological solution—administered directly into the stomach through the endoscope. The patient was positioned supine during the procedure, and 30-mL syringes were used for instillation. The volume of solution administered varied depending on the residual stomach size and the fluid loss toward the duodenum, ranging from 120 mL to 210 mL, with a median volume of 150 mL (Fig. 1).

All technical parameters for the CBCT acquisitions are summarized in Table 1. The dose-area product (DAP) for each CBCT acquisition was recorded and stored in the dose management software (Gray Detector, Elco).

**Table 1:** Technical parameters of upper abdomen CBCT acquisition using the Siemens ARTIS Pheno system (protocol “7 s Large Volume 360°”)

Parameter	Value
Tube voltage	90 kV*
Focus	Big
Pulse width	8 ms*
Dose	0.36 µGy/frame
Min. Cu-filter	0
Max. Cu-filter	0
Angulation step	0.80°/frame
Source–image distance	130 cm
Focus–isocenter distance	78.5 cm
Inherent filtration	2.5-mm aluminum equivalent
Field of view (height × width)	29.4 × 39.4 cm

* Values depend on body characteristics and vary during acquisition due to automatic exposure control.

The effective dose (ED) for the CBCT acquisitions was estimated using the PCXMC Rotation tool within the Monte Carlo software PCXMC v2.0 (STUK). For each patient, a simulation was performed in steps of 0.8°, up to a complete rotation of 360° for a total of 450 projections. Each simulation considered patient data (weight, height, age, position relative to the beam) and x-ray ergonomics parameters (irradiated field amplitude, kV, filtration, focus-reference point distance, DAP). The x-ray ergonomics parameters were extracted from the above-mentioned Gray Detector software. The equivalent dose for all organs was estimated by the software for each projection. The corresponding ED was calculated by means of ICRP 103 tissue weighting factors [15]. Total ED represents the sum of the 450 projections.

We referred to the European Guidelines to compare the dosimetric results from CBCT scans with the ED of conventional CT [16], which outline a standardized method for estimating ED in CT imaging. This involves multiplying the dose-length product (DLP) by a *k* coefficient specific to the anatomical region being scanned. For abdominal scans, *k* is 0.015 [17].

The Italian national dose reference level (DRL) for abdominal CT, corresponding to a DLP of 555 Gy·cm, served as the reference for comparison. [18].

### Gastric volume

The suitability of image quality for obtaining 3D stomach reconstructions and calculating gastric volume was evaluated by a radiologist with 5 years of experience in gastrointestinal imaging. The same radiologist performed the total gastric volume segmentation using the open-source 3D Slicer software (version 5.4.0, http://www.slicer.org). A slice-by-slice approach was employed, with volumetric regions of interest manually delineated to encompass the total gastric volume, including the stomach wall.

In scans acquired with CO₂ insufflation, the stomach appeared as an air-filled structure with very low attenuation, allowing clear identification of the gastric wall by its outer anatomical limits. In acquisitions performed after Gastrografin administration, the stomach was entirely filled with hyperdense contrast medium, facilitating the delineation of the stomach boundaries. In case of artifacts or ambiguous anatomical boundaries, segmentation was guided by anatomical landmarks and multiplanar reconstructions. Unclear regions were excluded from the final volumetric calculation.

**Fig. 2:**
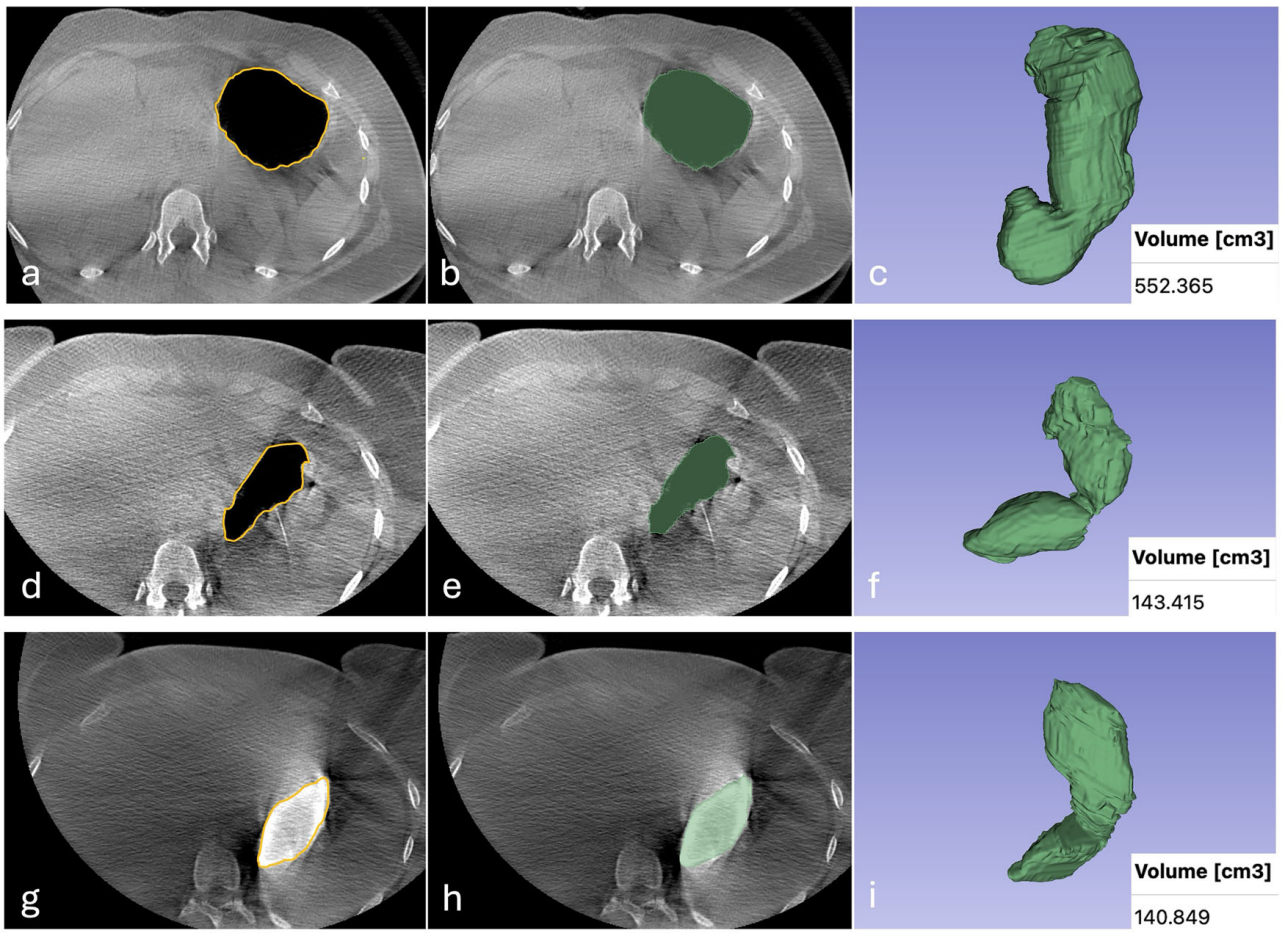
Upper abdomen CBCT images in a 45-year-old woman with a BMI of 32 kg/m^2^. Manually drawn regions of interest of the stomach with CO_2_ gastric insufflation before (**a**) and after ESG (**d**). Manually drawn region of interest of the stomach with administration of a diluted Gastrografin solution after ESG (**g**). The same images showing the stomach after volumetric segmentation (in green; **b**, **e**, **h**). The same stomach in 3D rendering (**c**, **f**, **i**). CBCT, Cone-beam computed tomography; ESG, Endoscopic sleeve gastroplasty

This process was performed across all CBCT scans acquired before and after ESG, with CO₂ gastric insufflation and Gastrografin administration (an example of the segmentation process is shown in Fig. 2). The software calculated the total gastric volume in cm³ based on the 3D segmentation by multiplying the number of image pixels by the pixel size and slice thickness.

### Statistical analysis

Data are given as median and interquartile range (IQR). Spearman’s correlation coefficient between BMI and ED was calculated for each scan type: pre- and post-ESG scans with CO₂ insufflation, and post-ESG scans with Gastrografin administration.

## Results

The study group consisted of 10 patients (7 female and 3 male), with a median age of 42.5 years (IQR: 41.3–48.5 years), a median body weight of 90.2 kg (IQR: 82.5–101.0 kg), and a median BMI of 34.8 kg/m² (IQR: 33.4–36.8 kg/m²).

A total of 30 CBCT scans were performed (two scans with CO_2_ insufflation respectively before and after ESG, one scan with administration of Gastrografin after ESG per patient). All CBCT scan images were of sufficient quality to obtain stomach 3D reconstructions and calculate gastric volumes. CBCT scans obtained with CO_2_ insufflation exhibited fewer artifacts and provided a more precise definition of the stomach wall than those acquired after Gastrografin administration (Fig. 1). The average time for manual segmentation was approximately 8 min per scan, depending on image quality, anatomical variability, and anatomical changes between pre- and post-procedural scans.

**Fig. 3:**
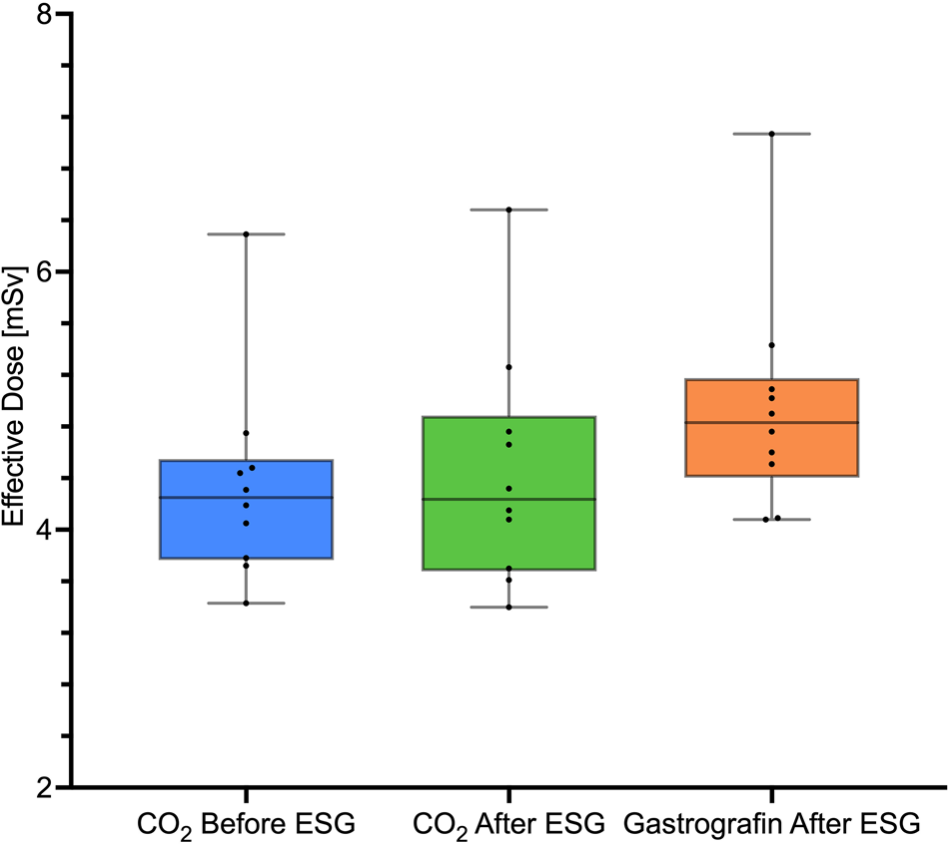
Boxplot of ED for the three different CBCT setup acquisitions. ESG, Endoscopic sleeve gastroplasty

The median ED for upper abdominal CBCT scans with CO_2_ insufflation was 4.2 mSv, both pre- and post-ESG (IQR: 3.8–4.5 mSv and 3.8–4.7 mSv, respectively). For post-ESG scans performed with Gastrografin administration, the median ED was 4.8 mSv (IQR: 4.5–5.1 mSv). Boxplots in Fig. 3 show the distribution of patients’ EDs for each acquisition setup. The ED estimated in all setups ranged from 3.4 mSv to 7.1 mSv, with an overall median ED of 4.5 mSv. Spearman correlation analysis revealed a statistically significant positive correlation between BMI and ED across all scan types. The correlation coefficients (ρ) were 0.818 for pre-ESG scans with CO_2_ insufflation (*p* = 0.004), 0.770 for post-ESG scans with CO_2_ insufflation (*p* = 0.009), and 0.855 for post-ESG scans with Gastrografin administration (*p* = 0.002).

Using the Italian national DRL, the reference ED for an abdominal CT scan (EDRef) is calculated to be 8.3 mSv. This value is approximately 50% higher than the median CBCT ED estimated in this study.

## Discussion

This preliminary study explores the feasibility of CBCT for gastric volumetry before and after ESG—an investigational approach not yet part of routine clinical practice, with no prior studies investigating its application for this clinical purpose.

Although the image quality was not comparable to conventional CT, all CBCT examinations demonstrated sufficient quality for stomach contouring and gastric volume calculations. CBCT imaging using CO_2_ insufflation resulted in fewer artifacts and a more precise representation of the stomach wall than scans performed after Gastrografin administration.

Given the risk of suture loss, ESG might not offer a permanent solution, but it has the advantage of being repeatable in cases of weight regain [19]. This makes monitoring gastric reservoir volume essential for patients undergoing ESG. A promising application of CBCT with CO_2_ insufflation under anesthesia is its ability to assess baseline gastric volume prior to ESG for procedure planning and to immediately evaluate post-procedural gastric volume and suture retention, all without requiring patient transport to a conventional CT scanner. For long-term follow-up, CBCT acquisitions with diluted Gastrografin could be performed without anesthesia, though its use for this purpose remains hypothetical and requires further validation.

Segmentation in this study was performed manually. Nonetheless, using alternative software—especially with automated or semiautomated tools—could greatly reduce processing times and improve clinical applicability.

An additional strength of this study is the use of the PCXMC rotational model to estimate the ED of upper abdominal CBCT scans in obese patients who had a mean BMI of 35.2 kg/m². The median ED values were approximately 50% of those associated with conventional CT, suggesting that CBCT may represent a valuable and potentially low-dose imaging modality for specific clinical indications. As CBCT is a relatively new imaging modality, limited research has focused on its radiation dose, with different approaches and modalities.

Li et al [20] assessed radiation exposure from abdominal CBCT scans during interventional procedures in 281 patients, with a mean BMI of 23.5 kg/m². The median DAP was 47.4 Gy·cm², with higher values associated with male gender and increased BMI. Sailer et al [21] evaluated radiation exposure in 40 patients with a mean BMI of 26.7 kg/m² undergoing endovascular interventions. Using a Monte Carlo simulation, the mean ED for upper abdominal CBCT was 4.9 mSv, with increased exposure linked to higher patient weight. Suzuki et al [22] aimed to evaluate the ED during abdominal CBCT scans on three different phantom sizes for three angiographic units. Using the Monte Carlo technique, ED of upper abdominal CBCT in a large-size phantom (BMI 29.3 kg/m²) was reported to be in the range from 2.6 mSv to 3.8 mSv, depending on the angiography unit’s CBCT technical parameters. Other studies reported the ED of abdominal CBCT to be in the range from 3.5 mSv to 25.4 mSv; however, these results are based on phantom studies or lack detailed information on the dose estimation method [23–27].

Furthermore, this study consistently found a positive correlation between BMI and ED across all setup acquisitions, with Spearman’s correlation coefficients ranging from 0.770 to 0.855 (all *p*-values < 0.01), indicating a moderate to strong association. These findings are in line with those of Dolenc et al [28], who reported a very strong positive association between BMI and ED in pelvic imaging (Spearman’s ρ = 0.888, *p* < 0.001).

Overall, by integrating quantitative dose assessment with an evaluation of image adequacy in a novel clinical context, this study supports the idea that CBCT may provide a practical compromise between radiation exposure and diagnostic effectiveness for gastric volumetry in obese patients undergoing ESG, highlighting its potential as a valuable imaging modality in this setting. Future studies could consider a direct comparison between CBCT and photon-counting CT (PCCT), which has demonstrated superior image quality and lower radiation exposure in other clinical contexts [29], in order to evaluate their relative performance in gastric volumetry. Nevertheless, CBCT retains practical advantages in the operating room, providing pre- and post-procedural volumetric imaging without the need for patient transport.

However, our investigation was a single-center study, with radiation dose data collected from obese patients within a defined BMI range undergoing ESG for bariatric purposes, which may introduce bias due to the targeted study population. Moreover, image analysis and gastric segmentation were performed by a single observer, and thus inter-observer agreement was not assessed. Furthermore, the sample size was small, and only one imaging protocol from a single angiographic system was used. Different manufacturers offer various settings that may result in different radiation doses. Therefore, future multicenter studies with larger patient populations and different CBCT systems are necessary to confirm the generalizability of these findings.

Although our results are promising, it is important to emphasize that CBCT for gastric volumetry remains an investigational technique, requiring further validation through extensive clinical research before widespread clinical adoption.

In conclusion, CBCT appears to be a feasible tool for gastric volumetric assessment at a relatively low radiation dose. Scans performed with CO_2_ gastric insufflation yield slightly lower radiation exposure and superior image quality than those using Gastrografin. As a result, CBCT could be a valuable tool for planning ESG and assessing suboptimal outcomes, both immediately after the procedure and during long-term follow-up.

## Supplementary information

**Additional file 1: Supplementary Fig. S1.** Endoscopic view of the gastric cavity prior to endoscopic sleeve gastroplasty (**a**). The gastric cavity in the body region immediately following completion of suturing (**b**).

## Data Availability

Data are available from the corresponding author upon reasonable request.

## Abbreviations

3D: Three-dimensional
BMI: Body mass index
CBCT: Cone-beam CT
DAP: Dose-area product
DLP: Dose-length product
ED: Effective dose
ESG: Endoscopic sleeve gastroplasty
IQR: Interquartile range
