# Supplementary material for: Low-dose cone-beam CT for gastric volumetry in endoscopic sleeve gastroplasty
Source: Eur Radiol Exp. 2025 Oct 7;9:98. doi: 10.1186/s41747-025-00637-3 (PMC12504165; doi:10.1186/s41747-025-00637-3)
Supplement: Supplementary file 1 — Additional file 1: Supplementary Fig. S1. Endoscopic view of the gastric cavity prior to endoscopic sleeve gastroplasty (a). The gastric cavity in the body region immediately following completion of suturing (b). [file 41747_2025_637_MOESM1_ESM.pdf]

# Low-dose cone-beam CT for gastric volumetry in endoscopic sleeve gastroplasty

## ELECTRONIC SUPPLEMENTARY MATERIAL

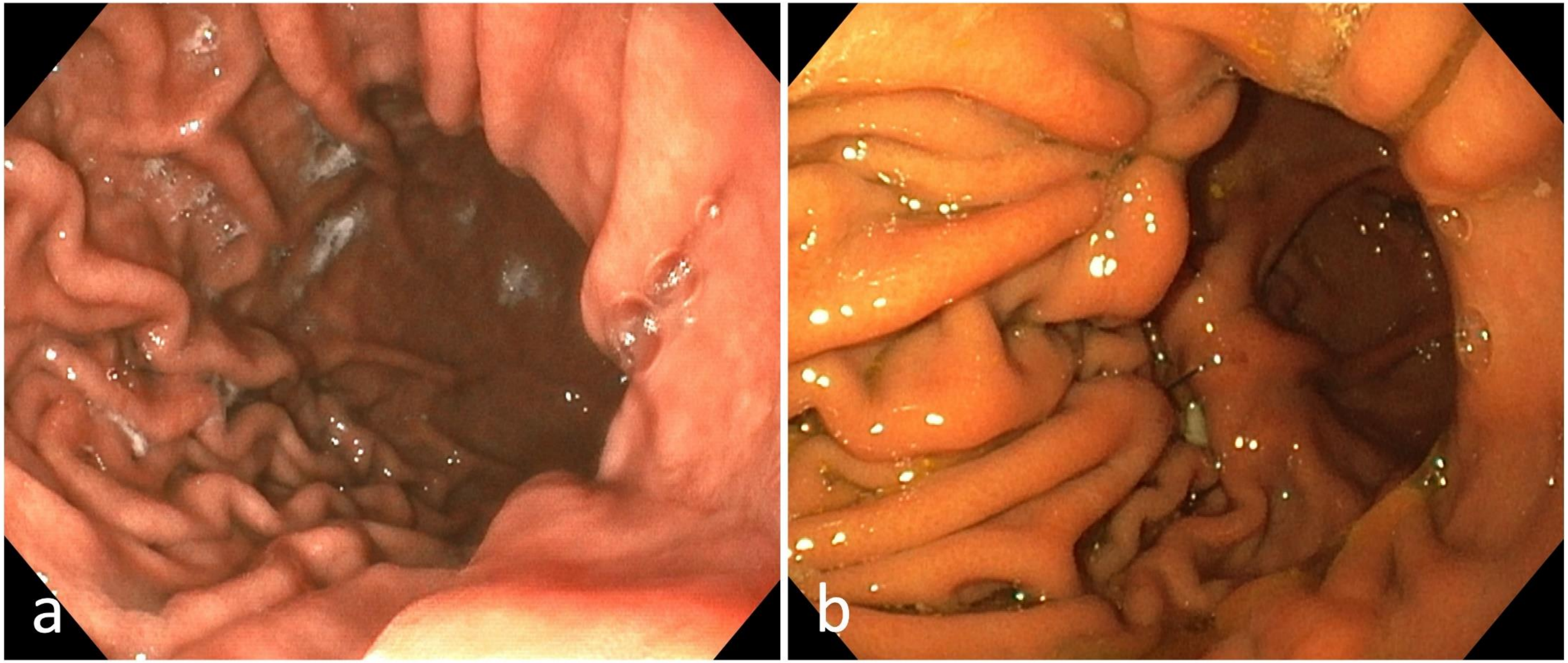


**Supplementary Fig. S1** Endoscopic view of the gastric cavity prior to endoscopic sleeve gastroplasty (**a**). The gastric cavity in the body region immediately following completion of suturing (**b**).
